# Angiogenesis inhibitor therapies for advanced renal cell carcinoma: Toxicity and treatment patterns in clinical practice from a global medical chart review

**DOI:** 10.3892/ijo.2013.2181

**Published:** 2013-11-15

**Authors:** WILLIAM K. OH, DAVID McDERMOTT, CAMILLO PORTA, ANTONIN LEVY, REZA ELAIDI, FLORIAN SCOTTE, ROBERT HAWKINS, DANIEL CASTELLANO, JOAQUIM BELLMUNT, SUN YOUNG RHA, JONG-MU SUN, PAUL NATHAN, BRUCE A. FEINBERG, JEFFREY SCOTT, RAY McDERMOTT, JIN-HEE AHN, JOHN WAGSTAFF, YEN-HWA CHANG, YEN-CHUAN OU, PAUL DONNELLAN, CHAO-YUAN HUANG, JOHN McCAFFREY, PO-HUI CHIANG, CHENG-KENG CHUANG, CAROLINE KORVES, MAUREEN P. NEARY, JOSE R. DIAZ, FAISAL MEHMUD, MEI SHENG DUH

**Affiliations:** 1 Dana-Farber Cancer Institute, Boston, MA;; 2 Mount Sinai School of Medicine, The Tisch Cancer Institute, New York, NY;; 3 Beth Israel Deaconess Medical Center, Boston, MA, USA;; 4 IRCCS San Matteo University Hospital Foundation, Pavia, Italy;; 5 Institut Gustave Roussy, Paris, France;; 6 Hôpital Européen Georges Pompidou, Paris, France;; 7 The Christie and University of Manchester, Manchester, UK;; 8 Unit of Uro-Oncology, Department of Medical Oncology, 12 de Octubre University Hospital, I+12 Research Institute, Madrid;; 9 Hospital del Mar - IMIM, Barcelona, Spain;; 10 Severance Hospital, Yonsei Cancer Center, Yonsei University College of Medicine, Seoul, Republic of Korea;; 11 Samsung Medical Center, Seoul, Republic of Korea;; 12 Mount Vernon Cancer Centre, Northwood, Middlesex, UK;; 13 P4 Healthcare, Ellicott City, MD, USA;; 14 Adelaide and Meath Hospital, Tallaght, Republic of Ireland;; 15 Asan Medical Center, Seoul, Republic of Korea;; 16 South West Wales Cancer Institute, Singleton Hospital, Swansea, UK;; 17 Taipei Veterans General Hospital, Taipei;; 18 Taichung Veterans General Hospital, Taichung, Taiwan, R.O.C.;; 19 University College Hospital Galway, Galway, Republic of Ireland;; 20 National Taiwan University Hospital, Taipei, Taiwan, R.O.C.;; 21 Mater Misericordiae Hospital, Dublin, Republic of Ireland;; 22 Chang Gung Memorial Hospital, Kaohsiung;; 23 Chang Gung Memorial Hospital, Linkuo, Taiwan, R.O.C.;; 24 Analysis Group Inc., Boston, MA;; 25 GlaxoSmithKline, Collegeville, PA, USA

**Keywords:** renal cell carcinoma, angiogenesis inhibitors, safety, treatment patterns, interruption, dose reduction

## Abstract

The aim of this study was to assess the treatment patterns and safety of sunitinib, sorafenib and bevacizumab in real-world clinical settings in US, Europe and Asia. Medical records were abstracted at 18 community oncology clinics in the US and at 21 tertiary oncology centers in US, Europe and Asia for 883 patients ≥18 years who had histologically/cytologically confirmed diagnosis of advanced RCC and received sunitinib (n=631), sorafenib (n=207) or bevacizumab (n=45) as first-line treatment. No prior treatment was permitted. Data were collected on all adverse events (AEs) and treatment modifications, including discontinuation, interruption and dose reduction. Treatment duration was estimated using Kaplan-Meier analysis. Demographics were similar across treatment groups and regions. Median treatment duration ranged from 6.1 to 10.7 months, 5.1 to 8.5 months and 7.5 to 9.8 months for sunitinib, sorafenib and bevacizumab patients, respectively. Grade 3/4 AEs were experienced by 26.0, 28.0 and 15.6% of sunitinib, sorafenib and bevacizumab patients, respectively. Treatment discontinuations occurred in 62.4 (Asia) to 63.1% (US) sunitinib, 68.8 (Asia) to 90.0% (Europe) sorafenib, and 66.7 (Asia) to 81.8% (US) bevacizumab patients. Globally, treatment modifications due to AEs occurred in 55.1, 54.2 and 50.0% sunitinib, sorafenib and bevacizumab patients, respectively. This study in a large, global cohort of advanced RCC patients found that angiogenesis inhibitors are associated with high rates of AEs and treatment modifications. Findings suggest an unmet need for more tolerable agents for RCC treatment.

## Introduction

Given that nearly 25% of all patients with kidney cancer present with locally advanced or metastatic renal cell carcinoma (RCC), kidney cancer is a malignancy with a poor prognosis (1). Conventional therapies, such as chemotherapy and radiation therapy are not effective and only 10 to 20% of patients benefit from immunotherapy (2–4). Recently enhanced understanding of the etiology of advanced RCC has led to the development of angiogenesis inhibitor agents.

In randomized clinical trials (RCTs), first generation angiogenesis inhibitors sunitinib, sorafenib and bevacizumab plus interferon α, have demonstrated efficacy in prolonging progression-free survival and/or overall survival as first-line treatment (4–6). The efficacy of sunitinib and sorafenib has also been established in expanded access programs (EAP) (7,8). Due to their strong efficacy profiles, these agents have become the new standard of treatment for advanced RCC. All these drugs were approved by corresponding regulatory agencies for use in the US, Europe and Asia (9–13).

However, RCTs and EAPs have also demonstrated that these angiogenesis inhibitors are associated with high rates of toxicity and treatment modifications, including discontinuations and dose changes. Since clinical trials may not be representative of real-life clinical practice due to treatment selection criteria, observational studies are necessary to understand the effects of treatment in the wider population of patients who actually receive these therapies. Small observational studies conducted in real-world clinical practice settings in US, Korea, Japan and Europe have provided further evidence of high toxicity profiles associated with these agents (14–21). Data from these varied care settings highlight that adverse events (AEs) in advanced RCC patients receiving angiogenesis therapies are common and often lead to treatment modifications, including treatment discontinuation.

As the use of angiogenesis inhibitors rises over time and treatment paradigms continue to evolve, there is a critical need to gain a thorough understanding of toxicity profiles and treatment patterns of these agents across various real-world clinical settings. Therefore, the goal of this study was to examine the toxicity profiles of sunitinib, sorafenib, and bevacizumab in advanced RCC among patients treated in US, Europe and Asia, and describe how clinicians in these settings modify treatment according to patient experiences.

## Materials and methods

### 

#### Study design

A retrospective study was conducted using data from medical records for eligible patients with advanced RCC who received anti-angiogenic therapies. The observation period for each patient started from the date of first angiogenesis inhibitor prescription or administration to the earliest of date of death, last follow-up date at the clinic or date of medical record abstraction. Data on second-line angiogenesis inhibitor treatment were also abstracted. The study drugs sunitinib, sorafenib and bevacizumab are manufactured by Pfizer, Bayer Healthcare Pharmaceuticals, and Hoffmann-LaRoche Inc., respectively.

#### Study population

To become eligible in the study patients were required to meet the following inclusion criteria: i) have had a confirmed histological and/or cytological diagnosis of locally advanced or metastatic RCC; ii) 18 years old or older at the time of confirmed diagnosis of advanced RCC; and iii) received at least 1 dose of oral sunitinib or sorafenib or intravenous (IV) administration of bevacizumab with or without interferon, after January 1, 2005. Previous immunotherapy or chemotherapy was not allowed. Patients were excluded if their first angiogenesis inhibitor treatment was initiated less than three months prior to the start date of medical record data abstraction, which varied across sites, to ensure adequate follow-up time.

#### Data source

Medical records for eligible patients were retrospectively abstracted by the clinical staff at 18 community oncology clinics in the US, and at 21 tertiary oncology centers across US (n=2), Europe (n=11; France n=2, Ireland n=3, Italy n=1, Spain n=2 and UK n=3), and Asia (n=8; Korea n=3 and Taiwan n=5). Data collected included date of RCC diagnosis, sociodemographic information, comorbidities, prior radiological treatments, metastatic site(s), baseline Eastern Cooperative Oncology Group (ECOG) performance status, dates and doses of anti-angiogenesis therapies prescribed or administered, reasons for changes in anti-angiogenesis therapies, and information on AEs. Other key data elements abstracted included the first and last dates of sunitinib, sorafenib and bevacizumab treatments, treatment modifications, and baseline and follow-up tumor measurements. Data were collected using a web-based case report form (CRF) created for this study. Data collection for this study spanned from July, 2007 through May, 2011. This study was approved in all centers by the ethics committees for tertiary oncology clinics and the New England Institutional Review Board for oncology community clinics in the US.

### Outcome definitions

#### Assessment of toxicity

All toxicity was analyzed retrospectively according to the experience recorded by investigators in daily clinical practice. AEs were graded using the National Cancer Institute Common Terminology Criteria for AEs (CTCAE) version 3.0. (22). If the severity of the AE was unknown then grade 1 was assigned. Only AEs experienced by patients during their first-line angiogenesis inhibitor treatment were considered for the assessment of safety.

#### Assessment of treatment patterns

Treatment modifications that occurred during first-line angiogenesis inhibitor treatment were examined. Reasons for treatment modifications were also abstracted from patients' medical records, if available. Treatment modifications considered were treatment discontinuation, treatment interruption (temporary stoppage of treatment with intent to resume treatment), dose reduction and dose increase. Patterns of switching between different angiogenesis inhibitors to second-line treatment were also examined, including reasons for switching.

#### Treatment duration

The duration of first-line treatment extended from the date of initiation of treatment to the date of treatment end, death, or last follow-up, whichever occurred first. Patients who did not discontinue their treatment were censored at the last follow-up.

#### Statistical analysis

Descriptive statistics were used to characterize baseline patient characteristics and report AE occurrences, and treatment patterns. Means and medians were used to describe continuous variables while frequencies and proportions were used to describe categorical variables. The Kaplan-Meier survival analysis method was used to calculate median treatment duration and account for censoring. The corresponding 95% confidence intervals (CI) were calculated using the log transformation method. All analyses were performed using SAS software version 9.2 (SAS Institute Inc., Cary, NC, USA).

## Results

### 

#### Patient characteristics

Table I presents the baseline characteristics of the patients. A total of 883 patients satisfied the eligibility criteria, including 157 (US), 349 (Europe), and 125 (Asia) patients treated with sunitinib; 131 (US), 60 (Europe), and 16 (Asia) patients treated with sorafenib; and 22 (US), 20 (Europe), and 3 (Asia) patients treated with bevacizumab. Most patients across the three geographical regions initiated treatment on recommended dosing: 50 mg QD 4/2 for sunitinib [range: 44.8% (Asia) to 84.8% (Europe)], 400 mg BID for sorafenib [range: 68.8% (Asia) to 80.0% (Europe)], and 10 mg/kg Q2WK for bevacizumab [range: 66.7% (Asia) to 91.0% (US)].

#### Toxicity profile

Table II presents the rates of all grade and grade 3/4 AEs. The proportion of sunitinib patients experiencing at least one AE was about 87% across all regions. Among patients receiving sorafenib, 78.3% (Europe) to 87.8% (US) experienced at least one AE, and among patients receiving bevacizumab, 33.3% (Asia) to 77.3% (US) experienced at least one AE. Specific AEs experienced by at least 5% of patients in at least one treatment group are reported. The three most common all grade AEs in patients treated with sunitinib were fatigue/asthenia [range: 18.4% (Asia) to 58.5% (Europe)], mucositis/stomatitis [range: 22.9% (US) to 42.1% (Europe)] and diarrhea [range: 17.6% (Asia) to 34.4% (US)]. Patients treated with sorafenib commonly experienced the following all grade AEs: fatigue/asthenia [range: 6.3% (Asia) to 39.7% (US)], diarrhea [range: 6.7% (Asia) to 35.1% (US)], and nausea [range: 5.0% (Europe) to 23.7% (US)]. Among patients who received bevacizumab, the most common all grade AEs reported on the medical charts were fatigue/asthenia [up to 45.4% (US)] and proteinuria [up to 22.7% (US)].

#### Treatment patterns

Table III summarizes treatment patterns for first-line treatment with angiogenesis inhibitor. Median treatment duration, in months, was 6.1 (US), 10.7 (Europe) and 10.7 (Asia) for sunitinib; 5.1 (US), 8.5 (Europe) and 7.1 (Asia) for sorafenib; and 9.2 (US), 9.8 (Europe) and 7.5 (Asia) for bevacizumab. Treatment discontinuation occurred in 62.4% (Asia) to 63.1% (US) of patients treated with sunitinib, 68.8% (Asia) to 90.0% (Europe) of patients treated with sorafenib, and 66.7% (Asia) to 81.8% (US) of patients treated with bevacizumab. Reasons for treatment modifications were available at all but one site. Among sites with these data, progressive disease was the most commonly recorded reason for treatment discontinuation [sunitinib, 33.1% (US) to 40.0% (Asia); sorafenib, 42.0% (US) to 55.6% (Europe); bevacizumab, 33.3% (Asia) to 46.7% (Europe)] followed by AEs [sunitinib, 18.4% (Asia) to 23.6% (US); sorafenib, 6.3% (Asia) to 28.2% (US); bevacizumab, 0% (Asia) to 27.3% (US)]. Drug dosage was reduced in 34.4% (US) to 48.0% (Asia) of patients treated with sunitinib, and 21.7% (Europe) to 45.8% (US) of patients treated with sorafenib. AEs were the most commonly reported reason for reduced dosage for sunitinib [29.2% (US), 37.1% (Europe), and 36.8% (Asia)], and sorafenib [43.5% (US), 20.4% (Europe), and 31.3% (Asia)]. After the discontinuation of first-line treatment, 20.4% (US) to 24.0% (Asia) of patients treated with sunitinib, 31.3% (Asia) to 50.0% (Europe) of patients treated with sorafenib, and 59.1% (US) to 70.0% (Europe) of patients treated with bevacizumab received second-line therapy.

Table IV describes specific AEs reported as reasons for first-line treatment modifications. Among the AEs of interest, vomiting was the most common AE leading to discontinuation of first-line sunitinib in the US (21.6%), fatigue/asthenia was most common in Europe (32.7%), and mucositis or stomatitis was the most common reason in Asia (34.8%). For sorafenib, skin rash most commonly led to treatment discontinuation in the US (27.8%); diarrhea and hand-foot syndrome most commonly led to discontinuation in Europe (37.5%). The most common AEs reported as reason for dose reduction for sunitinib varied across regions: diarrhea in US (25.5%), fatigue in Europe (31.5%) and mucositis or stomatitis in Asia (26.1%). Skin rash was the most common AE for dose reduction among sorafenib patients in US (31.6%), and hand-foot syndrome was the most common reason in Europe (44.4%).

## Discussion

Findings from the current study with data from 883 patients contribute to the growing body of knowledge on the use of sunitinib, sorafenib and bevacizumab as first-line agents among patients with advanced RCC treated in real-world clinical practice across different geographical regions. Tolerability and management of side-effects for patients receiving sunitinib, sorafenib and bevacizumab as first-line anti-angiogenesis treatment for advanced RCC are significant issues for patients and the physicians who care for them.

There were variations in treatment and outcomes across global regions. In the US and Europe, patients receiving sunitinib were almost twice as likely as those in Asia to initiate therapy at 50 mg QD 4/2. This observation raises the issue relative to the use of fixed doses of suntinib (and also of all molecularly targeted agents), irrespective of parameters like gender and body weight/body surface area. Indeed, a population pharmacokinetic analysis identified low body weight (and female gender) as covariates that significantly increase exposure to sunitinib, potentially leading to increased toxicity (23). Moreover, the most commonly experienced AEs also varied across region. In the US and Europe, fatigue was the most common AE among patients receiving sunitinib, sorafenib and bevacizumab while hand-foot syndrome was the most common AE in Asian patients receiving sunitinib or sorafenib. Notably, the AEs most commonly leading to treatment discontinuation also varied.

Despite these differences, there were some universal findings across settings. Notably, the median treatment duration was generally shorter in this observational study compared with that reported in RCTs and EAPs. The median treatment duration for sunitinib was 11 and 16.6 months in the RCT and EAP (4,7), respectively, whereas the median ranged from 6.1 to 10.7 months across regions in this study. Similarly, the median treatment duration for sorafenib was 12 months in the EAP (8) whereas it ranged from 5.1 to 8.5 months across regions in the current analysis. For bevacizumab the median treatment duration was 9.7 months in one RCT (24) and 8.2 months in another RCT (25) whereas it ranged from 7.5 to 9.8 months across regions in this study.

Treatment discontinuation was high, reaching 63.1% among sunitinib patients in the US, 90.0% among sorafenib patients in Europe, and 81.8% of bevacizumab patients in US. For patients who discontinued sunitinib treatment due to AEs the average number of AEs per discontinuation was 2.3–2.5 across regions; for sorafenib it was 1.9–3.1. This illustrates that in real-world practice physicians manage multiple AEs per patient and may discontinue or modify treatment based on the observed effects of these AEs. The proportion of patients with any type of treatment modification due to an AE was also consistently high across all regions, reaching 59.2% among sunitinib patients in Asia, 64.1% among sorafenib patients in US and 53.3% of bevacizumab patients in Europe.

This study builds upon prior evidence from RCTs and EAPs on the toxicity and treatment patterns of angiogenesis inhibitors. Similar to results in this global chart review study, treatment discontinuation was high in RCTs and EAPs. In the RCT comparing sunitinib to interferon α, 86% of patients experienced a treatment discontinuation (versus 62.4 to 63.1% in the current study) (4). In the EAP for sorafenib, 100% of patients experienced a treatment discontinuation (versus 68.8 to 90.0% in the current study) (8). In the RCT comparing bevacizumab plus interferon to placebo, 72% of patients experienced a treatment discontinuation (versus 66.7 to 81.8% in the current study) (24). In RCTs, diarrhea was the most commonly reported AE for both sunitinib and sorafenib (versus fatigue in the US and Europe, and hand-foot syndrome in Asia in the current study) and anorexia was the most commonly reported AE for bevacizumab (versus fatigue in the current study for US and Europe) (4,8,24). In EAPs for sunitinib and sorafenib, the most commonly reported AEs were diarrhea and hand-foot syndrome.

Differences with RCT may arise due to differences in the underlying study populations as well as how data are collected. For example, the proportion of patients with brain metastasis in the current study were 7.0, 7.2 and 11.1%, for sunitinib, sorafenib and bevacizumab, respectively, while these patients were excluded in RCTs. Besides, RCTs have well-defined operational definitions for AE identification and gradation, as well as a rigorous protocol to capture them whereas in a retrospective setting such as in the current study, AEs are captured based on the treating physicians' reports and judgments. Sometimes physicians may record only those AEs that lead to a treatment modification or if the AEs are severe enough to warrant specific treatment; therefore, under-reporting of AEs in this study may have occurred.

Findings from this study were generally consistent with those from other observational studies (14–20). In the Korean study by Hong *et al* (14), 76% of sunitinib patients had a dose interruption or dose reduction due to AEs, and 11% overall discontinued due to toxicity. A high proportion of patients in that study (>75%) experienced fatigue, anorexia and hand-foot syndrome. In the Korean study by Hwang *et al* (15), 29% of sunitinib patients experienced a dose reduction. In the UK study by Ansari *et al* (16), 15% of sunitinib patients experienced a dose discontinuation in their first cycle of treatment, and 75% experienced at least one dose reduction. Notably, the number of patients in the current study was several fold higher than the aforementioned observational studies.

Some disparities in study results between this study and other observational studies reported above may have occurred due to differences in treatment durations, frequency of patient visits where AEs are reported, incomplete or inadequate recording of AEs, and differences in practice patterns relative to management of AEs across countries. Differences in drug approval dates, affecting drug availability, could have affected practice patterns as well. Differences in healthcare should also be kept in mind while making comparisons across studies.

There are some limitations associated with this study. Since data collection for this study preceded marketing authorization for pazopanib in Europe, this study does not include information on patients receiving pazopanib as first-line treatment. Further, due to the small sample sizes in certain groups for some regions, especially bevacizumab in all regions and sorafenib in Asia, the findings reported are descriptive in nature.

This multi-country study provides evidence that AEs are common in patients with advanced RCC treated with angiogenesis inhibitors, and that these AEs often lead to treatment modifications in the real-world clinical setting. This real-world practice study suggests that management of toxicities associated with anti-angiogenic agents for the treatment of advanced RCC presents significant issues for treating physicians and patients. The findings from this study further underscore the continued need for novel tolerable treatment options for advanced RCC. Additionally, the results of this study show the potential benefits of use of observational studies to further understand real-world treatment patterns and outcomes, beyond information that may be available from other data sources.

## Figures and Tables

**Table I.** Baseline clinical characteristics among patients with advanced RCC receiving first-line angiogenesis inhibitor treatment.^a^

	United States			Europe			Asia		
	SU (n=157)	SOR (n=131)	BEV (n=22)	SU (n=349)	SOR (n=60)	BEV (n=20)	SU (n=125)	SOR (n=16)	BEV (n=3)
Initial dose, n (%)	50 mg QD 4/2 127 (80.9) 37.5 mg QD (4/2) 4 (2.5) 25 mg QD (4/2) 7 (4.5) Other 16 (10.2) Unknown 3 (1.9)	800 mg BID 1 (0.8) 400 mg BID 97 (74.0) 200 mg BID 25 (19.1) Other 8 (6.1)	10 mg/kg Q2WK 20 (91.0)5 mg/kg Q2WK 1 (4.5) Unknown 1 (4.5)	50 mg QD 4/2 296 (84.8) 37.5 mg QD 4/2 29 (8.3) Other 14 (4.0) Unknown 10 (2.9)	800 mg BID 4 (6.7) 400 mg BID 48 (80.0) Other 5 (8.3) Unknown 3 (5.0)	11 mg/kg Q2WK 1 (5.0) 10 mg/kg Q2WK 16 (80.0) Unknown 3 (15.0)	50 mg QD 4/2 56 (44.8) 37.5 mg QD 4/2 11 (8.8) 25 mg QD 4/2 1 (0.8) Other 57 (45.6)	800 mg BID 3 (18.8) 400 mg BID 11 (68.8) Other 2 (12.5)	10 mg/kg Q2WK 2 (66.7) Unknown 1 (33.3)
Age at TI (years)									
Median (range)	62.3 (29.7–93.3)	65.6 (26.0–88.0)	61.1 (41.9–86.8)	62.0 (23.3–88.6)	63.0 (27.2–86.3)	61.1 (45.5–76.2)	57.3 (24.3–86.1)	54.3 (46.2–80.4)	55.5 (35.4–78.1)
Mean (SD)	63.7 (11.6)	65.7 (11.3)	60.7 (12.3)	61 (11.7)	62 (12.1)	60 (8.5)	58 (13.5)	57 (10.2)	56 (21.3)
Male, n (%)	102 (65.0)	82 (62.6)	16 (72.7)	240 (68.8)	47 (78.3)	15 (75.0)	102 (81.6)	11 (68.8)	2 (66.7)
ECOG PS, n (%)									
0	27 (17.2)	26 (19.8)	4 (18.2)	108 (30.9)	20 (33.3)	7 (35.0)	19 (15.2)	2 (12.5)	0 (0.0)
1	41 (26.1)	41 (31.3)	7 (31.8)	74 (21.2)	7 (11.7)	5 (25.0)	48 (38.4)	6 (37.5)	2 (66.7)
2	5 (3.2)	12 (9.2)	3 (13.6)	19 (5.4)	2 (3.3)	1 (5.0)	10 (8.0)	1 (6.3)	0 (0.0)
3	1 (0.6)	1 (0.8)	0 (0.0)	3 (0.9)	0 (0.0)	0 (0.0)	2 (1.6)	0 (0.0)	0 (0.0)
4	0 (0.0)	0 (0.0)	0 (0.0)	2 (0.6)	0 (0.0)	0 (0.0)	0 (0.0)	0 (0.0)	0 (0.0)
Unknown	83 (52.9)	51 (38.9)	0 (0.0)	143 (41.0)	31 (51.7)	7 (35.0)	46 (36.8)	7 (43.8)	1 (33.3)
No. of MS, n (%)									
0	9 (5.7)	2 (1.3)	0 (0.0)	16 (4.6)	3 (5.0)	0 (0.0)	1 (0.8)	1 (6.3)	1 (33.3)
1	63 (40.1)	68 (43.3)	7 (31.8)	156 (44.7)	28 (46.7)	5 (25.0)	76 (60.8)	11 (68.8)	1 (33.3)
2	50 (31.8)	39 (24.8)	7 (31.8)	100 (28.7)	17 (28.3)	8 (40.0)	35 (28.0)	2 (12.5)	0 (0.0)
>2	33 (21.0)	18 (11.5)	7 (31.8)	76 (21.8)	12 (20.0)	7 (35.0)	13 (10.4)	2 (12.5)	1 (33.3)
Unknown	2 (1.3)	4 (2.5)	1 (4.5)	1 (0.3)	0 (0.0)	0 (0.0)	0 (0.0)	0 (0.0)	0 (0.0)
Metastatic sites, n (%)									
Lung	88 (56.1)	77 (58.8)	12 (54.5)	225 (64.5)	36 (60.0)	15 (75.0)	83 (66.4)	10 (62.5)	1 (33.3)
Lymph nodes	40 (25.5)	18 (13.7)	6 (27.3)	111 (31.8)	21 (35.0)	8 (40.0)	21 (16.8)	2 (12.5)	1 (33.3)
Liver	16 (10.2)	19 (14.5)	5 (22.7)	70 (20.1)	8 (13.3)	3 (15.0)	17 (13.6)	3 (18.8)	0 (0.0)
Bone	57 (36.3)	40 (30.5)	9 (40.9)	74 (21.2)	12 (20.0)	5 (25.0)	34 (27.2)	4 (25.0)	1 (33.3)
Brain	16 (10.2)	10 (7.6)	3 (13.6)	23 (6.6)	3 (5.0)	2 (10.0)	5 (4.0)	2 (12.5)	0 (0.0)
Time from initial RCC diagnosis to treatment (months)									
Median (range)	4.4 (0.1–339.3)	6.4 (0.1–480.8)	21.6 (1.2–86.1)	9.8 (0.0–222.4)	21.3 (0.5–158.4)	13.8 (1.1–101.7)	9.4 (0.0–222.6)	3.8 (0.3–83.8)	9.0 (6.6–39.1)
<1 year, n (%)	99 (63.1)	76 (58.0)	10 (45.5)	186 (53.3)	25 (41.7)	10 (50.0)	68 (54.4)	11 (68.8)	2 (66.7)
Prior therapy, n (%)									
Nephrectomy	114 (72.6)	89 (67.9)	18 (81.8)	242 (69.3)	50 (83.3)	18 (90.0)	97 (77.6)	12 (75.0)	2 (66.7)
Radiation therapy	36 (22.9)	33 (25.2)	11 (50.0)	23 (6.6)	10 (16.7)	2 (10.0)	7 (5.6)	1 (6.3)	0 (0.0)
Comorbidities, n (%)									
Hypertension	43 (27.4)	41 (31.3)	10 (45.5)	66 (18.9)	31 (51.7)	4 (20.0)	42 (33.6)	4 (25.0)	0 (0.0)
Diabetes	3 (1.9)	4 (3.1)	2 (9.1)	17 (5.8)	10 (18.5)	0 (0.0)	16 (12.8)	3 (18.8)	0 (0.0)

BEV, bevacizumab; ECOG, Eastern Cooperative Oncology Group; BID, two times a day; QD, once a day; Q2WK, once per 2 weeks; RCC, renal cell carcinoma; SOR, sorafenib; SD, standard deviation; SU, sunitinib; TI, treatment initiation; PS, performance score; MS, metastatic sites.

a Observations were made during the baseline period, defined as the period up to the initiation of first-line angiogenesis inhibitor treatment. For variables with multiple assessments over time (ECOG performance score), the last available assessment during the baseline period was reported.

**Table II.** Adverse events by severity among patients with advanced RCC receiving first-line angiogenesis inhibitor treatment.^a^

	United States			Europe			Asia		
	SU (n=157)	SOR (n=131)	BEV (n=22)	SU (n=349)	SOR (n=60)	BEV (n=20)	SU (n=125)	SOR (n=16)	BEV (n=3)
Patients with at least one adverse event, n (%)									
All grades	137 (87.3)	115 (87.8)	17 (77.3)	302 (86.5)	47 (78.3)	13 (65.0)	111 (88.8)	13 (81.3)	1 (33.3)
Grades 3 and 4	46 (29.3)	41 (31.3)	3 (13.6)	84 (24.1)	11 (18.3)	4 (20.0)	34 (27.2)	6 (37.5)	0 (0.0)
Specific adverse events, n (%)^b^									
Abdominal pain									
All grades	11 (7.0)	10 (7.6)	0 (0.0)	4 (1.1)	0 (0.0)	0 (0.0)	3 (2.4)	1 (6.3)	0 (0.0)
Grades 3 and 4	1 (0.6)	0 (0.0)	0 (0.0)	0 (0.0)	0 (0.0)	0 (0.0)	1 (0.7)	1 (6.3)	0 (0.0)
Alopecia									
All grades	1 (0.6)	10 (7.6)	0 (0.0)	8 (2.3)	0 (0.0)	1 (5.0)	2 (1.6)	1 (6.3)	0 (0.0)
Grades 3 and 4	0 (0.0)	0 (0.0)	0 (0.0)	0 (0.0)	0 (0.0)	0 (0.0)	0 (0.0)	0 (0.0)	0 (0.0)
Anemia									
All grades	18 (11.5)	5 (3.8)	0 (0.0)	10 (2.9)	1 (1.7)	0 (0.0)	10 (8.0)	1 (6.3)	0 (0.0)
Grades 3 and 4	0 (0.0)	0 (0.0)	0 (0.0)	1 (0.3)	0 (0.0)	0 (0.0)	2 (1.6)	0 (0.0)	0 (0.0)
Anorexia									
All grades	26 (16.6)	17 (13.0)	5 (22.7)	47 (13.5)	5 (8.3)	1 (5.0)	24 (19.2)	1 (6.3)	0 (0.0)
Grades 3 and 4	0 (0.0)	2 (1.5)	0 (0.0)	1 (0.3)	0 (0.0)	0 (0.0)	0 (0.0)	0 (0.0)	0 (0.0)
Constipation									
All grades	13 (8.3)	10 (7.6)	2 (9.1)	25 (7.2)	2 (3.3)	0 (0.0)	8 (6.4)	0 (0.0)	0 (0.0)
Grades 3 and 4	1 (0.6)	0 (0.0)	0 (0.0)	0 (0.0)	0 (0.0)	0 (0.0)	0 (0.0)	0 (0.0)	0 (0.0)
Cough									
All grades	4 (2.5)	4 (3.1)	1 (4.5)	10 (2.9)	0 (0.0)	0 (0.0)	10 (8.0)	1 (6.3)	0 (0.0)
Grades 3 and 4	0 (0.0)	0 (0.0)	0 (0.0)	0 (0.0)	0 (0.0)	0 (0.0)	0 (0.0)	0 (0.0)	0 (0.0)
Decreased taste sensation									
All grades	15 (9.6)	4 (3.1)	0 (0.0)	24 (6.9)	0 (0.0)	0 (0.0)	2 (1.6)	0 (0.0)	0 (0.0)
Grades 3 and 4	1 (0.6)	0 (0.0)	0 (0.0)	0 (0.0)	0 (0.0)	0 (0.0)	0 (0.0)	0 (0.0)	0 (0.0)
Dehydration									
All grades	11 (7.0)	6 (4.6)	0 (0.0)	3 (0.9)	0 (0.0)	0 (0.0)	0 (0.0)	0 (0.0)	0 (0.0)
Grades 3 and 4	1 (0.6)	0 (0.0)	0 (0.0)	1 (0.3)	0 (0.0)	0 (0.0)	0 (0.0)	0 (0.0)	0 (0.0)
Diarrhea									
All grades	54 (34.4)	46 (35.1)	4 (18.2)	119 (34.1)	4 (6.7)	2 (10.0)	22 (17.6)	3 (18.8)	0 (0.0)
Grades 3 and 4	4 (2.5)	5 (3.8)	0 (0.0)	8 (2.3)	0 (0.0)	0 (0.0)	1 (0.8)	1 (6.3)	0 (0.0)
Dyspnea									
All grades	18 (11.5)	8 (6.1)	1 (4.5)	26 (7.4)	1 (1.7)	1 (5.0)	6 (4.8)	3 (18.8)	0 (0.0)
Grades 3 and 4	4 (2.5)	1 (0.8)	0 (0.0)	3 (0.9)	0 (0.0)	0 (0.0)	0 (0.0)	0 (0.0)	0 (0.0)
Edema (any location)									
All grades	11 (7.0)	1 (0.8)	0 (0.0)	17 (4.9)	0 (0.0)	1 (5.0)	14 (11.2)	0 (0.0)	0 (0.0)
Grades 3 and 4	0 (0.0)	0 (0.0)	0 (0.0)	2 (0.6)	0 (0.0)	0 (0.0)	1 (0.8)	0 (0.0)	0 (0.0)
Epistaxis									
All grades	0 (0.0)	0 (0.0)	0 (0.0)	11 (3.2)	0 (0.0)	0 (0.0)	3 (2.4)	0 (0.0)	0 (0.0)
Grades 3 and 4	0 (0.0)	0 (0.0)	0 (0.0)	1 (0.3)	0 (0.0)	0 (0.0)	0 (0.0)	0 (0.0)	0 (0.0)
Fatigue or asthenia									
All grades	72 (45.9)	52 (39.7)	10 (45.4)	204 (58.5)	21 (35.0)	4 (20.0)	23 (18.4)	1 (6.3)	0 (0.0)
Grades 3 and 4	8 (5.1)	5 (3.8)	1 (4.5)	26 (7.4)	4 (6.7)	1 (5.0)	1 (0.8)	0 (0.0)	0 (0.0)
Fever and/or chills									
All grades	15 (9.6)	6 (4.6)	0 (0.0)	3 (0.9)	0 (0.0)	0 (0.0)	9 (7.2)	1 (6.3)	0 (0.0)
Grades 3 and 4	1 (0.6)	0 (0.0)	0 (0.0)	2 (0.6)	0 (0.0)	0 (0.0)	3 (2.0)	0 (0.0)	0 (0.0)
Hand-foot syndrome									
All grades	20 (12.7)	33 (25.2)	0 (0.0)	91 (26.1)	6 (10.0)	0 (0.0)	49 (39.2)	6 (37.5)	0 (0.0)
Grades 3 and 4	1 (0.6)	8 (6.1)	0 (0.0)	10 (2.9)	1 (1.7)	0 (0.0)	6 (4.8)	2 (12.5)	0 (0.0)
Hemorrhage									
All grades	9 (5.7)	7 (5.3)	1 (4.5)	7 (2.0)	0 (0.0)	0 (0.0)	2 (1.6)	0 (0.0)	0 (0.0)
Grades 3 and 4	0 (0.0)	2 (1.5)	0 (0.0)	1 (0.3)	0 (0.0)	0 (0.0)	1 (0.8)	0 (0.0)	0 (0.0)
Hypertension									
All grades	20 (12.7)	12 (9.2)	4 (18.2)	50 (14.3)	3 (5.0)	1 (5.0)	21 (16.8)	1 (6.3)	1 (33.3)
Grades 3 and 4	0 (0.0)	2 (1.5)	0 (0.0)	0 (0.0)	1 (1.7)	0 (0.0)	1 (0.8)	0 (0.0)	0 (0.0)
Hypothyroidism									
All grades	1 (0.6)	0 (0.0)	0 (0.0)	17 (4.9)	0 (0.0)	0 (0.0)	7 (5.6)	0 (0.0)	0 (0.0)
Grades 3 and 4	0 (0.0)	0 (0.0)	0 (0.0)	0 (0.0)	0 (0.0)	0 (0.0)	0 (0.0)	0 (0.0)	0 (0.0)
Mucositis or stomatitis									
All grades	36 (22.9)	18 (13.7)	2 (9.1)	147 (42.1)	4 (6.7)	1 (5.0)	46 (36.8)	3 (18.8)	0 (0.0)
Grades 3 and 4	4 (2.5)	6 (4.6)	0 (0.0)	13 (3.7)	0 (0.0)	0 (0.0)	4 (3.2)	0 (0.0)	0 (0.0)
Nausea									
All grades	43 (27.4)	31 (23.7)	4 (18.2)	74 (21.2)	3 (5.0)	1 (5.0)	14 (11.2)	2 (12.5)	0 (0.0)
Grades 3 and 4	3 (1.9)	2 (1.5)	0 (0.0)	3 (0.9)	0 (0.0)	0 (0.0)	0 (0.0)	0 (0.0)	0 (0.0)
Oral pain									
All grades	2 (1.3)	2 (1.5)	0 (0.0)	16 (4.6)	0 (0.0)	0 (0.0)	5 (4.0)	0 (0.0)	0 (0.0)
Grades 3 and 4	0 (0.0)	0 (0.0)	0 (0.0)	0 (0.0)	0 (0.0)	0 (0.0)	0 (0.0)	0 (0.0)	0 (0.0)
Other skin problems^c^									
All grades	16 (10.2)	16 (12.2)	0 (0.0)	38 (10.9)	2 (3.3)	0 (0.0)	1 (0.8)	1 (6.3)	0 (0.0)
Grades 3 and 4	0 (0.0)	0 (0.0)	0 (0.0)	0 (0.0)	1 (1.7)	0 (0.0)	0 (0.0)	0 (0.0)	0 (0.0)
Pain									
All grades	29 (18.5)	27 (20.6)	2 (9.1)	56 (16.0)	0 (0.0)	3 (15.0)	23 (18.4)	3 (18.8)	0 (0.0)
Grades 3 and 4	4 (2.5)	5 (3.8)	0 (0.0)	4 (1.1)	0 (0.0)	0 (0.0)	4 (3.2)	0 (0.0)	0 (0.0)
Proteinuria									
All grades	0 (0.0)	0 (0.0)	5 (22.7)	3 (0.9)	0 (0.0)	2 (10.0)	2 (1.6)	0 (0.0)	0 (0.0)
Grades 3 and 4	0 (0.0)	0 (0.0)	3 (13.6)	0 (0.0)	0 (0.0)	1 (5.0)	1 (0.8)	0 (0.0)	0 (0.0)
Skin rash									
All grades	19 (12.1)	50 (38.2)	2 (9.1)	37 (10.6)	5 (8.3)	2 (10.0)	26 (20.8)	4 (25.0)	0 (0.0)
Grades 3 and 4	1 (0.6)	9 (6.9)	0 (0.0)	4 (1.1)	1 (1.7)	0 (0.0)	0 (0.0)	1 (6.3)	0 (0.0)
Thrombocytopenia									
All grades	13 (8.3)	3 (2.3)	0 (0.0)	15 (4.3)	1 (1.7)	0 (0.0)	14 (11.2)	0 (0.0)	0 (0.0)
Grades 3 and 4	5 (3.2)	0 (0.0)	0 (0.0)	3 (0.9)	0 (0.0)	0 (0.0)	2 (1.6)	0 (0.0)	0 (0.0)
Urinary problems^d^									
All grades	6 (3.8)	9 (6.9)	0 (0.0)	1 (0.3)	0 (0.0)	0 (0.0)	0 (0.0)	1 (6.3)	0 (0.0)
Grades 3 and 4	0 (0.0)	1 (0.8)	0 (0.0)	0 (0.0)	0 (0.0)	0 (0.0)	0 (0.0)	0 (0.0)	0 (0.0)
Vomiting									
All grades	24 (15.3)	16 (12.2)	1 (4.5)	27 (7.7)	1 (1.7)	1 (5.0)	6 (4.8)	2 (12.5)	0 (0.0)
Grades 3 and 4	1 (0.6)	2 (1.5)	0 (0.0)	1 (0.3)	0 (0.0)	0 (0.0)	0 (0.0)	0 (0.0)	0 (0.0)
Weight loss									
All grades	8 (5.1)	12 (9.2)	0 (0.0)	1 (0.3)	0 (0.0)	0 (0.0)	3 (2.4)	0 (0.0)	0 (0.0)
Grades 3 and 4	1 (0.6)	0 (0.0)	0 (0.0)	0 (0.0)	0 (0.0)	0 (0.0)	0 (0.0)	0 (0.0)	0 (0.0)

BEV, bevacizumab; RCC, renal cell carcinoma; SOR, sorafenib; SD, standard deviation; SU, sunitinib.

a Grade 1 severity was assumed for adverse events with unknown severity.

b Adverse events experienced by at least 5% of patients in at least one treatment group are reported.

c Other skin problems include yellow, orange or generally discolored skin, erythematous lesion, cracking, aching, red spots on the forehead, facial skin lesions, dry skin and desquamation of skin.

d Urinary problems include burning or frequent urination, urinary retention, dysuria, nocturia, urinary tract infection and urosepsis.

**Table III.** Treatment patterns among patients with advanced RCC receiving first-line angiogenesis inhibitor treatment

Variable	United States			Europe			Asia		
	SU (n=157)	SOR (n=131)	BEV (n=22)	SU (n=349)	SOR (n=60)	BEV (n=20)	SU (n=125)	SOR (n=16)	BEV (n=3)
First-line treatment									
Patients who discontinued first-line treatment, n (%)	99 (63.1)	103 (78.6)	18 (81.8)	220 (63.0)	54 (90.0)	16 (80.0)	78 (62.4)	11 (68.8)	2 (66.7)
Duration of treatment^a^									
Median (95% CI)^b,c^	6.1 (5.1–7.1)	5.1 (3.9–6.0)	9.2 (6.0–18.9)	10.7 (9.1–12.7)	8.5 (7.2–14.0)	9.8 (7.3–11.0)	10.7 (7.0–14.2)	7.1 (2.4–12.1)	7.5 (6.6–7.2)
Mean (SD)	7.8 (0.5)	8.1 (0.8)	13.7 (2.4)	17.4 (1.1)	15.0 (1.8)	24.0 (13.8)	8.4 (7.5)	4.7 (3.4)	6.9 (0.4)
Reason for discontinuation, n (%)^d,e^									
Progressive disease	52 (33.1)	55 (42.0)	10 (45.5)	107 (36.8)	30 (55.6)	7 (46.7)	50 (40.0)	7 (43.8)	1 (33.3)
Adverse events	37 (23.6)	37 (28.2)	6 (27.3)	55 (18.9)	8 (14.8)	3 (20.0)	23 (18.4)	1 (6.3)	0 (0.0)
Complete response	0 (0.0)	0 (0.0)	0 (0.0)	1 (0.3)	0 (0.0)	0 (0.0)	0 (0.0)	0 (0.0)	0 (0.0)
Stable disease	0 (0.0)	0 (0.0)	0 (0.0)	1 (0.3)	1 (1.9)	0 (0.0)	0 (0.0)	0 (0.0)	0 (0.0)
Other	16 (10.2)	17 (13.0)	2 (9.1)	10 (3.4)	2 (3.7)	0 (0.0)	6 (4.8)	0 (0.0)	1 (33.3)
Unknown	0 (0.0)	0 (0.0)	0 (0.0)	17 (5.8)	10 (18.5)	2 (13.3)	3 (2.4)	3 (18.8)	0 (0.0)
Patients with first-line treatment interruption, n (%)^e^	49 (31.2)	57 (43.5)	5 (22.7)	88 (30.2)	8 (14.8)	3 (20.0)	40 (32.0)	6 (37.5)	1 (33.3)
Reason for treatment interruption, n (%)^d,e^									
Adverse event	44 (28.0)	51 (38.9)	5 (22.7)	68 (23.4)	8 (14.8)	3 (20.0)	35 (28.0)	4 (25.0)	1 (33.3)
Surgery	0 (0.0)	0 (0.0)	0 (0.0)	6 (2.1)	1 (1.9)	0 (0.0)	2 (1.6)	1 (6.3)	0 (0.0)
Other	13 (8.3)	8 (6.1)	1 (4.5)	6 (2.1)	0 (0.0)	0 (0.0)	3 (2.4)	0 (0.0)	0 (0.0)
Unknown	0 (0.0)	0 (0.0)	0 (0.0)	10 (3.4)	0 (0.0)	0 (0.0)	4 (3.2)	1 (6.3)	0 (0.0)
Patients with first-line treatment dose increase, n (%)^8^	18 (11.5)	26 (19.8)	0 (0.0)	44 (12.6)	5 (8.3)	1 (5.0)	19 (15.2)	2 (12.5)	0 (0.0)
Reason for dose increase, n (%)^d,e^									
Good tolerance	4 (2.5)	6 (4.6)	0 (0.0)	18 (6.2)	1 (1.9)	0 (0.0)	0 (0.0)	0 (0.0)	0 (0.0)
Symptom improvement	0 (0.0)	0 (0.0)	0 (0.0)	0 (0.0)	0 (0.0)	0 (0.0)	3 (2.4)	0 (0.0)	0 (0.0)
Adverse events improved	1 (0.5)	3 (2.3)	0 (0.0)	0 (0.0)	0 (0.0)	0 (0.0)	0 (0.0)	0 (0.0)	0 (0.0)
Adverse events	0 (0.0)	0 (0.0)	0 (0.0)	7 (2.4)	0 (0.0)	0 (0.0)	4 (3.2)	0 (0.0)	0 (0.0)
Progressive disease	0 (0.0)	4 (3.1)	0 (0.0)	4 (1.4)	1 (1.9)	0 (0.0)	1 (0.8)	1 (6.3)	0 (0.0)
Other	14 (8.9)	16 (12.2)	0 (0.0)	6 (2.1)	1 (1.9)	0 (0.0)	0 (0.0)	0 (0.0)	0 (0.0)
Unknown	0 (0.0)	0 (0.0)	0 (0.0)	7 (2.4)	3 (5.6)	0 (0.0)	0 (0.0)	0 (0.0)	0 (0.0)
Patients with first-line treatment dose reduction, n (%)^8^	54 (34.4)	60 (45.8)	0 (0.0)	150 (43.0)	13 (21.7)	3 (15.0)	60 (48.0)	6 (37.5)	0 (0.0)
Reason for dose reduction, n (%)^d,e^									
Adverse events	47 (29.9)	57 (43.5)	0 (0.0)	108 (37.1)	11 (20.4)	3 (20.0)	46 (36.8)	5 (31.3)	0 (0.0)
Other	11 (7.0)	5 (3.8)	0 (0.0)	7 (2.4)	0 (0.0)	0 (0.0)	6 (4.8)	1 (6.3)	0 (0.0)
Unknown	0 (0.0)	0 (0.0)	0 (0.0)	9 (3.1)	2 (3.7)	0 (0.0)	10 (8.0)	0 (0.0)	0 (0.0)
Patients who experienced at least one treatment modification, n (%) ^f^	136 (86.6)	118 (90.1)	19 (86.4)	250 (85.9)	51 (94.4)	15 (100.0)	106 (84.8)	14 (87.5)	3 (100.0)
Patients who experienced at least one treatment modification due to adverse events, n (%)^e,f^	88 (56.1)	84 (64.1)	11 (50.0)	154 (52.9)	18 (33.3)	8 (53.3)	74 (59.2)	7 (43.8)	1 (33.3)
Second- and third-line treatment									
Patients who received second-line treatment, n (%)	32 (20.4)	60 (45.8)	13 (59.1)	79 (22.6)	30 (50.0)	14 (70.0)	30 (24.0)	5 (31.3)	2 (66.7)
Reason for first-line treatment discontinuation, n (%)^d,e^									
Progressive disease	20 (12.7)	40 (30.5)	6 (27.3)	46 (15.8)	17 (31.5)	0 (0.0)	23 (18.4)	2 (12.5)	1 (33.3)
Adverse events	12 (7.6)	21 (16.0)	5 (22.7)	18 (6.2)	2 (3.7)	0 (0.0)	5 (4.0)	1 (6.3)	0 (0.0)
Surgery	0 (0.0)	0 (0.0)	0 (0.0)	1 (0.3)	0 (0.0)	0 (0.0)	2 (1.6)	0 (0.0)	0 (0.0)
Other	1 (0.6)	3 (2.3)	2 (9.1)	1 (0.3)	0 (0.0)	0 (0.0)	2 (1.6)	0 (0.0)	1 (33.3)
Unknown	0 (0.0)	0 (0.0)	0 (0.0)	3 (1.0)	9 (16.7)	0 (0.0)	0 (0.0)	2 (12.5)	0 (0.0)

BEV, bevacizumab; CI, confidence interval; NR, not reached; RCC, renal cell carcinoma; SOR, sorafenib; SD, standard deviation; SU, sunitinib.

a Patients who died on the day of discontinuation were not counted as having discontinued treatment.

b The Kaplan-Meier survival analysis method was used to account for censoring.

c Patients who did not have an event (discontinuation) were censored at the end of follow-up.

d For patients with more than one distinct reason for treatment modification, each distinct reason for modification is included.

e Information regarding treatment interruption and reasons for treatment modifications were not available from one site in Europe. Hence, the denominators for calculation of proportions for reasons of treatment modifications and proportion of treatment interruption in the pan-European region are sunitinib (n=291), sorafenib (n=54) and bevacizumab (n=15).

f Treatment modification includes treatment discontinuation, treatment interruption, dose increase and dose reduction.

**Table IV.** Adverse events reported as reasons for treatment modifications among patients with advanced RCC receiving first-line angiogenesis inhibitor treatment.

	United States			Europe^c^			Asia		
		
	SU (n=157)	SOR (n=131)	BEV (n=22)	SU (n=291)	SOR (n=54)	BEV (n=15)	SU (n=125)	SOR (n=16)	BEV (n=3)
Treatment discontinuation									
Patients who discontinued first-line treatment due to adverse events, n (%)	37 (23.6)	36 (27.5)	8 (36.4)	55 (18.9)	8 (14.8)	3 (20.0)	23 (18.4)	1 (6.3)	0 (0.0)
Adverse events per discontinuation, mean (SD)	2.3 (1.3)	1.9 (1.0)	1.5 (0.7)	2.5 (1.1)	3.1 (1.4)	3 (1.7)	2.5 (0.7)	2.0 (N/A)	N/A
Adverse events resulting in a treatment discontinuation, n (%)^a,b^									
Diarrhea	5 (13.5)	2 (5.6)	0 (0.0)	5 (9.1)	3 (37.5)	1 (33.3)	0 (0.0)	0 (0.0)	0 (0.0)
Fatigue or asthenia	6 (16.2)	8 (22.2)	0 (0.0)	18 (32.7)	2 (25.0)	1 (33.3)	2 (8.7)	0 (0.0)	0 (0.0)
Hand-foot syndrome	4 (10.8)	4 (11.1)	0 (0.0)	5 (9.1)	3 (37.5)	0 (0.0)	5 (21.7)	0 (0.0)	0 (0.0)
Hypertension	2 (5.4)	2 (5.6)	0 (0.0)	3 (5.5)	1 (12.5)	0 (0.0)	2 (8.7)	0 (0.0)	0 (0.0)
Mucositis or stomatitis	0 (0.0)	0 (0.0)	0 (0.0)	5 (9.1)	1 (12.5)	0 (0.0)	8 (34.8)	0 (0.0)	0 (0.0)
Nausea	7 (18.9)	6 (16.7)	0 (0.0)	2 (3.6)	0 (0.0)	0 (0.0)	0 (0.0)	0 (0.0)	0 (0.0)
Skin rash	4 (10.8)	10 (27.8)	1 (12.5)	3 (5.5)	1 (12.5)	0 (0.0)	1 (4.3)	1(100.0)	0 (0.0)
Vomiting	8 (21.6)	4 (11.1)	0 (0.0)	4 (7.3)	0 (0.0)	1 (33.3)	0 (0.0)	0 (0.0)	0 (0.0)
Dose interruption									
Patients with first-line treatment dose interruption due to adverse events, n (%)	44 (28.0)	51 (38.9)	5 (22.7)	68 (23.4)	8 (14.8)	3 (20.0)	35 (28.0)	4 (25.0)	1(33.3)
Adverse events per interruption, mean (SD)	2.0 (1.4)	1.7 (1.3)	1.2 (0.5)	2.9 (1.3)	2.4 (0.7)	2.7 (1.2)	2.6 (1.0)	2.5 (0.6)	2.0 (0.0)
Adverse events resulting in a treatment interruption, n (%)^a,b^									
Diarrhea	5 (11.4)	9 (17.6)	0 (0.0)	15 (22.1)	1 (12.5)	0 (0.0)	2 (5.7)	0 (0.0)	0 (0.0)
Fatigue or asthenia	15 (34.1)	2 (3.9)	0 (0.0)	18 (26.5)	2 (25.0)	0 (0.0)	5 (14.3)	0 (0.0)	0 (0.0)
Hand-foot syndrome	6 (13.6)	8 (15.7)	0 (0.0)	7 (10.3)	2 (25.0)	0 (0.0)	8 (22.9)	2 (50.0)	0 (0.0)
Hypertension	4 (9.1)	5 (9.8)	1 (20.0)	2 (2.9)	0 (0.0)	0 (0.0)	3 (8.6)	0 (0.0)	0 (0.0)
Mucositis or stomatitis	4 (9.1)	10 (19.6)	0 (0.0)	17 (25.0)	1 (12.5)	1 (33.3)	1 (2.9)	0 (0.0)	0 (0.0)
Nausea	7 (15.9)	5 (9.8)	0 (0.0)	6 (8.8)	0 (0.0)	0 (0.0)	1 (2.9)	1 (25.0)	0 (0.0)
Skin rash	1 (2.3)	17 (33.3)	0 (0.0)	6 (8.8)	3 (37.5)	0 (0.0)	1 (2.9)	0 (0.0)	0 (0.0)
Vomiting	5 (11.4)	4 (7.8)	0 (0.0)	10 (14.7)	0 (0.0)	0 (0.0)	1 (2.9)	1 (25.0)	0 (0.0)
Dose reduction									
Patients with first-line treatment dose reduction due to adverse events, n (%)	47 (29.9)	57 (43.5)	0 (0.0)	108 (37.1)	9 (16.7)	3 (20.0)	46 (36.8)	5 (18.8)	0 (0.0)
Adverse events per reduction, mean (SD)	2.1 (1.2)	1.7 (1.2)	N/A	3.3 (1.4)	3.1 (1.9)	4.0 (2.0)	2.6 (0.8)	2.4 (0.9)	N/A
Adverse events of interest resulting in a dose reduction, n (%)^a,b^									
Diarrhea	12 (25.5)	13 (22.8)	0 (0.0)	27 (25.0)	2 (22.2)	0 (0.0)	7 (15.2)	1 (20.0)	0 (0.0)
Fatigue or asthenia	10 (21.3)	7 (12.3)	0 (0.0)	34 (31.5)	3 (33.3)	1 (33.3)	3 (6.5)	0 (0.0)	0 (0.0)
Hand-foot syndrome	3 (6.4)	14 (24.6)	0 (0.0)	19 (17.6)	4 (44.4)	0 (0.0)	10 (21.7)	3 (60.0)	0 (0.0)
Hypertension	7 (14.9)	6 (10.5)	0 (0.0)	9 (8.3)	0 (0.0)	0 (0.0)	4 (8.7)	1 (20.0)	0 (0.0)
Mucositis or stomatitis	9 (19.1)	10 (17.5)	0 (0.0)	32 (29.6)	3 (33.3)	1 (33.3)	12 (26.1)	0 (0.0)	0 (0.0)
Nausea	8 (17.0)	4 (7.0)	0 (0.0)	18 (16.7)	1 (11.1)	0 (0.0)	0 (0.0)	0 (0.0)	0 (0.0)
Skin rash	1 (2.1)	18 (31.6)	0 (0.0)	14 (13.0)	1 (11.1)	0 (0.0)	3 (6.5)	2 (40.0)	0 (0.0)
Vomiting	3 (6.4)	2 (3.5)	0 (0.0)	13 (12.0)	1 (11.1)	0 (0.0)	2 (4.3)	0 (0.0)	0 (0.0)

BEV, bevacizumab; RCC, advanced renal cell carcinoma; SOR, sorafenib; SD, standard deviation; SU, sunitinib.

a Patients may have experienced more than one adverse event leading to a treatment modification.

b This table presents only the adverse events of interest that were associated with treatment modifications. Other adverse events that resulted in treatment modifications are not listed here.

c Information regarding treatment interruption and reasons for treatment modifications were not available from one site in Europe. Hence, the denominators for calculation of proportions for reasons of treatment modifications in the pan-European region are sunitinib (n=291), sorafenib (n=54) and bevacizumab (n=15).
